# Additive-manufactured Ti-6Al-4 V/Polyetheretherketone composite porous cage for Interbody fusion: bone growth and biocompatibility evaluation in a porcine model

**DOI:** 10.1186/s12891-021-04022-0

**Published:** 2021-02-11

**Authors:** Pei-I Tsai, Meng-Huang Wu, Yen-Yao Li, Tzu-Hung Lin, Jane S. C. Tsai, Hsin-I Huang, Hong-Jen Lai, Ming-Hsueh Lee, Chih-Yu Chen

**Affiliations:** 1 Biomedical Technology and Device Research Laboratories, Industrial Technology Research Institute, Chutung, Hsinchu, Taiwan; 2 Department of Orthopedics, Taipei Medical University Hospital, Taipei, Taiwan; 3 Department of Orthopaedics, School of Medicine, College of Medicine, Taipei Medical University, Taipei, Taiwan; 4 Department of Orthopedic Surgery, Chang Gung Memorial Hospital, Chiayi, Taiwan; 5 College of Medicine, Chang Gung University, Taoyuan, Taiwan; 6 Material and Chemical Research Laboratories, Industrial Technology Research Institute, Chutung, Hsinchu, Taiwan; 7 Material and Chemical Research Laboratories, Industrial Technology Research Institute, Hsinchu, 31040 Taiwan; 8 Department of Neurosurgery, Department of Surgery, Chang Gung Memorial Hospital, Chiayi, 61363 Taiwan; 9 Department of Nursing, Chang Gung University of Science and Technology, Chiayi, Taiwan; 10 Department of Orthopedics, Shuang-Ho Hospital, Taipei Medical University, No.291, Zhongzheng Rd., Zhonghe District, New Taipei City, 23561 Taiwan

**Keywords:** Additive manufacturing (3D printing), Ti, 6Al, 4 V (Ti alloy)/polyetheretherketone (PEEK) composite porous cage, porcine study

## Abstract

**Background:**

We developed a porous Ti alloy/PEEK composite interbody cage by utilizing the advantages of polyetheretherketone (PEEK) and titanium alloy (Ti alloy) in combination with additive manufacturing technology.

**Methods:**

Porous Ti alloy/PEEK composite cages were manufactured using various controlled porosities. Anterior intervertebral lumbar fusion and posterior augmentation were performed at three vertebral levels on 20 female pigs. Each level was randomly implanted with one of the five cages that were tested: a commercialized pure PEEK cage, a Ti alloy/PEEK composite cage with nonporous Ti alloy endplates, and three composite cages with porosities of 40, 60, and 80%, respectively. Micro-computed tomography (CT), backscattered-electron SEM (BSE-SEM), and histological analyses were performed.

**Results:**

Micro-CT and histological analyses revealed improved bone growth in high-porosity groups. Micro-CT and BSE-SEM demonstrated that structures with high porosities, especially 60 and 80%, facilitated more bone formation inside the implant but not outside the implant. Histological analysis also showed that bone formation was higher in Ti alloy groups than in the PEEK group.

**Conclusion:**

The composite cage presents the biological advantages of Ti alloy porous endplates and the mechanical and radiographic advantages of the PEEK central core, which makes it suitable for use as a single implant for intervertebral fusion.

**Supplementary Information:**

The online version contains supplementary material available at 10.1186/s12891-021-04022-0.

## Background

Spinal fusion is a surgical treatment modality for several spinal diseases. Over an estimated 400,000 spinal fusions are performed annually in the United States, and approximately 2.8 million spinal fusions were conducted from 2004 to 2015 [1]. Spinal fusion is the standard treatment for 96% of patients with degenerative spinal diseases in the United States [2]. This surgery is intended to restore intervertebral disc height and achieve bony fusion between them. Several surgical procedures and fusion devices have been developed to achieve high fusion rates and optimize clinical outcomes.

Interbody techniques effectively restore disc height and lead to improved fusion rates [3]. Interbody cage technology for spinal fusion was first proposed by Bagby in 1988 [4]. Interbody cages have been designed as a space holder implanted between adjacent bony endplates and allows bone to grow through the space in order to achieve osseous integration of adjacent vertebral bodies [5–7]. A report noted that among patients undergoing spinal fusion surgery for degenerative spondylolisthesis, as many as 83% of procedures involved the use of an interbody cage [8].

Several materials have been used to manufacture interbody cages, of which polyetheretherketone (PEEK) and titanium (Ti) alloy are the most commonly used [7] because of their excellent biocompatibility [9]. Ti and its alloys have been extensively used in the orthopedic industry since the 1940s [10]. Ti demonstrates excellent corrosion resistance in a clinical environment and is widely used in additive manufacturing technology [7, 11, 12]. Its ability to enhance cell adhesion and osseointegration is a crucial advantage for implanted orthopedic devices [9, 10]. However, Ti cages are radiopaque; therefore, evaluating the bone fusion status over time through standard clinical imaging modalities by clinicians becomes difficult [13]. Furthermore, because of their high elastic modulus, Ti cages result in high implant subsidence rates [7, 14, 15].

PEEK is a hydrophobic polymer with a low elastic modulus; thus, it can reduce the possibility of subsidence into spinal endplates. PEEK cages were introduced in the 1990s [16]. Unlike Ti cages, implanted PEEK cages are radiolucent; however, their hydrophobic surface property makes protein absorption unlikely, resulting in poor cell adhesion and bone growth [17]. Animal studies have found that PEEK cages are encapsulated by a thin fibrous tissue layer, which can inhibit implant–host bone growth, resulting in pseudarthrosis, nonfusion, implant migration, and subsidence [7, 18].

Therefore, an ideal cage design should have the material advantages of both Ti and PEEK to achieve high implant-bone affinity, facilitate bone growth, bone similar biomechanical characters, and radiolucency for fusion mass observation. Several studies have reported the use of a composite material cage with both Ti and PEEK [7, 12, 19]. However, no study has evaluated a porous structure with different porosity rates.

We manufactured an innovative Ti-6Al-4 V (Ti alloy)/PEEK composite porous cage through laser grooving, plasma spraying, and additive manufacturing (selective laser melting [SLM]) technology. By using the previously developed laser grooving and plasma spraying technologies [20], the shear bonding strength between Ti alloy and the PEEK interface could be significantly increased. Moreover, using additive manufacturing technology, we could easily obtain different porosity rates at the endplate Ti alloy layer to facilitate bone ingrowth and strong fusion constructs. We hypothesized that this porous composite cage design facilitates bone fusion and leads to stronger fusion constructs compared with standard PEEK, Ti alloy, or nonporous Ti alloy/PEEK composite cages. The second objective is to identify the best porosity rate suitable for bone growth.

## Methods

### Production of the additive-manufactured Ti-6Al-4 V/PEEK composite porous cage

**Fig. 1:**
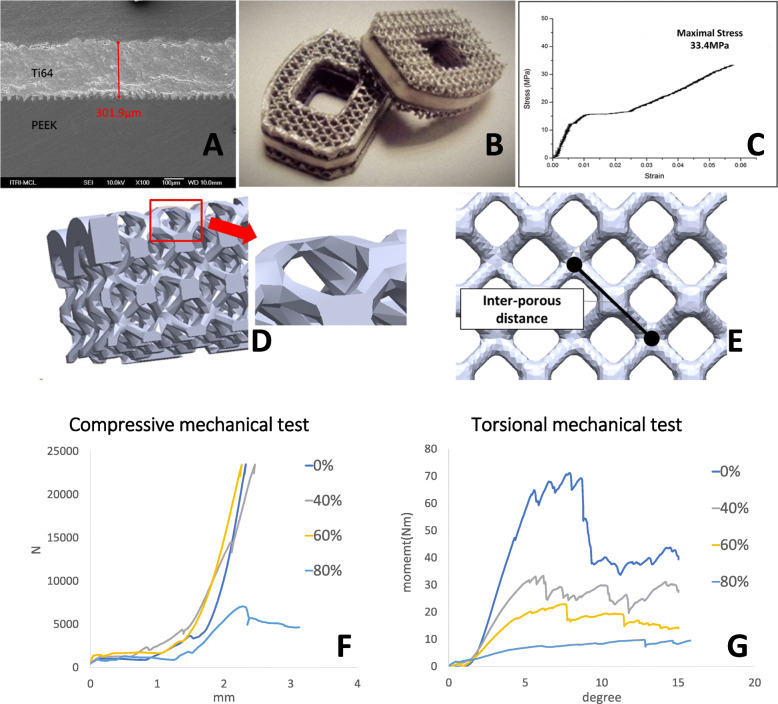
Fabrication of the additive-manufactured Ti-6Al-4 V (Ti alloy)/polyetheretherketone (PEEK) composite porous cage. **a** Surface modification through laser grooving and plasma spraying makes the interfacial layer thicker than 300 μm. **b** Finished product of the Ti alloy/PEEK composite porous cage. **c** Shear strength of the bonding interface between the metallic layer and PEEK substrate exceeded 30 MPa. d Schematic of the porous structure with inset. **e** Schematic of the interporous distance. **f** Results of the mechanical compression test on the composite porous cage. **g** Results of the mechanical torsion test on the composite porous cage

The innovative composite porous interbody cage was produced using the polymer core material PEEK, which is a favorable biomedical material with high strength and toughness. The surface of the PEEK polymer substrate was modified through laser grooving to enhance the bonding strength of the subsequent coating layer (Fig. 1a and b). The surface was then coated with a metallic (Ti alloy) interfacial layer through low-temperature arc ion plating and plasma spraying to ensure sufficient thickness of the metallic interface layer (300 μm).

Subsequently, a 3-mm-thick 3D Ti-6Al-4 V porous scaffold was constructed above the metallic interfacial layer with intended porosity parameters by using the SLM EOSINT M 270 model (EOS GambH-Electro Optical Systems, Krailling, Germany).

The microstructure of the metallic interfacial layer was observed through multifunctional field-emission scanning electron microscopy (SEM), and its chemical composition was analyzed through SEM with energy-dispersive spectroscopy (SEM-EDS). The shear strength of the bonding interface between the metallic layer and PEEK substrate exceeded 30 MPa (Fig. 1c).

The results of the compressive mechanical test on the composite porous interbody cage are shown in Fig. 1f and Table 1, and those of the torsional mechanical test are shown in Fig. 1g and Table 2.

**Table 1:** Results of the compressive mechanical test on the composite porous interbody cage

Porosity rate (%)	Yield load (N)	Stiffness (N/mm)
0	> 23,000	35,420.96
40	> 23,000	30,831.72
60	> 23,000	29,654.86
**80**	**7047.09**	**7374.72**

**Table 2:** Results of the torsional mechanical test on the composite porous interbody cage

Porosity rate (%)	Stiffness (Nm/degree)	Yield moment (Nm)
0	7.12	41.61
40	3.59	26.37
60	2.47	19.66
80	1.63	11.13

The porosity rate was defined using the following equation:

$$ P\%=\left(1-\frac{D_{i\_ porous}}{D_{solid}}\right)\ast 100\% $$

where *P*% is the porosity rate, *D*_*i_porous*_ is the measured density of the metallic layer (measured mass divided by measured volume), and *D*_*solid*_ is the density of the metallic layer in the solid condition. The parameters of different porosity rates are listed in Table 3. Schematics of the porous structure and interporous distance are illustrated in Fig. 1d and e. Based on these parameters, composite cages with metallic layers of different porosities were produced. The geometric parameters of metallic and composite cages in this study were derived from the commercialized PEEK cage (Anterior Cervical Interbody Fusion Cage®, BAUI biotech, New Taipei City, Taiwan).

**Table 3:** Unit porous size, interporous distance, total volume, and total surface area of the cage at different porosities

Porosity rate (%)	Unit porous size (*mm*^3^)	Interporous distance (*mm*)	Total volume of cage materials (*mm*^3^)	Total surface area (*mm*^2^)	Young’s modulus (GPa)
0	Nil	Nil	600	631	1.53
40	0.23	0.40	380	2391	1.33
60	0.23	0.31	300	2406	1.28
80	0.23	0.22	190	2137	0.32

### Study design

This animal study was approved by the Ethics Committee of the Biomedical Technology and Device Research Laboratories of Industrial Technology Research Institute in accordance with National Animal Welfare Legislation (Approval No. PIG-1040106), and the study protocol conformed to the National Institute of Health guidelines for the use of laboratory animals. Twenty 5-month-old female pigs (Lanyu 50, Taiwan) from different litters, weighing 35–45 kg, were used in this study following Zou et al.’s protocol [21]. All the pigs were obtained commercially from PigModel Animal Technology Co., Ltd. (Miaoli, Taiwan). Each pig underwent anterior intervertebral lumbar fusion at three levels: L2–L3, L4–L5, and L6–L7. Each level was randomly implanted with one of the five test cages. Each of the five groups comprised 12 specimens. In the first group, we tested a commercialized pure PEEK interbody device with an autologous iliac crest bone graft (PEEK_NonP, group 1). In the second group, a Ti alloy/PEEK composite cage with nonporous Ti alloy endplates embedded with an autologous iliac crest bone graft was tested (Comp_NonP, group 2). The third, fourth, and fifth groups used composite cages embedded with an autologous iliac crest bone graft with porosities of 40, 60%, or 80% on both Ti alloy endplates (Comp_40%P [group 3], Comp_60%P [group 4], and Comp_80%P [group 5]). Each fusion segment was additionally secured with pedicle screws (Lumbar Trans-Pedicle Screw Fixation System®; Wiltrom Biotech Co., Ltd., New Taipei City, Taiwan) in implanted levels. All pigs were kept in single pens throughout the 6-month observation period and were subsequently euthanized. Tetracycline (20 mg/kg; SIGMA-Aldrich, Merck Group, Germany) was injected intravenously at 4 and 2 weeks before euthanasia to label bone growth.

The pigs were euthanized under deep anesthesia with intravenously injected KCl (1–2 mEq/kg). Plain radiographs of anteroposterior and lateral views and computed tomography (CT) of the lumbosacral spine were taken at euthanasia. The whole lumbar spinal column from L1 to L7 was removed en bloc, stripped of the soft tissue, transported to the laboratory, and stored at − 20 °C for further examination. The pigs were bred for scientific purposes and handled according to the regulation of the Institutional Animal Care and Use Committee (IACUC: PIG-106022) on animal experimentation.

### Surgical methods

Before anesthesia, the pigs were premedicated intramuscularly with 5 mg/kg Zoletil 50 (Zolazepam + Tiletamine) + 2.2 mg/kg Xylazine for induction. After orotracheal intubation, anesthesia was maintained through the inhalation of isoflurane (1.5%). Cephalosporin (1 g, intravenously) was administered 30 min before surgery as a prophylactic antibiotic.

Under aseptic conditions, the autologous bone graft was harvested from the right iliac crest with the pig placed in a prone position and prepared as morselized cancellous bone chips. Under fluoroscopic control, the intervertebral space in implanted levels was identified before surgical intervention. The facet joints of the neighboring vertebrae at this level were exposed through a posterior midline incision and paraspinal bilateral intramuscular approach. Pedicle screws (5 mm in diameter and 30 mm in length) were inserted into the neighboring vertebrae transpedicularly. The incision in the back was carefully sutured and the pigs were closely cared for 1 month to allow complete recovery. After the pigs’ condition become stable, we performed the 2nd stage operation. With the pigs placed in the left decubitus position, a retroperitoneal anterior approach was used. The rectus abdominis muscle and its sheath were incised and retracted. The innermost layer, the fascia of transverse abdominis, was carefully dissected to prevent damage to the peritoneum lying immediately underneath. After the peritoneum and its contents were separated and retracted, the quadratus lumborum and psoas major muscles could be viewed. The anterior lumbar spine was easily identified by its thick and shiny anterior longitudinal ligament. After the ligation and cutting of segmental vessels, the L2–L3, L4–L5, and L6–L7 intervertebral discs were excised together with the cranial and caudal endplates, ring apophysis, and part of the anterior longitudinal ligament. Thereafter, the bone graft was morselized and packed into the central holes of the respective interbody cage devices. The fusion device–bone graft complexes were then implanted at each intervertebral disc. After insertion of the three implants, the abdominal muscles and the rectus abdominis sheath were carefully sutured, and the skin was closed using running sutures. Prophylactic cephalosporin (1.0 g, intravenously) and analgesic ketorolac (30 mg, intramuscularly) were administered before and immediately after surgery. All pigs were kept in individual pens and fed a normal diet containing 1.4% calcium and 0.7% phosphorus (percent of food weight). Pain control medication was administered for 7 days postoperatively (400 mg ibuprofen, two tablets/day) and as required afterward.

### Micro-CT analysis

**Fig. 2:**
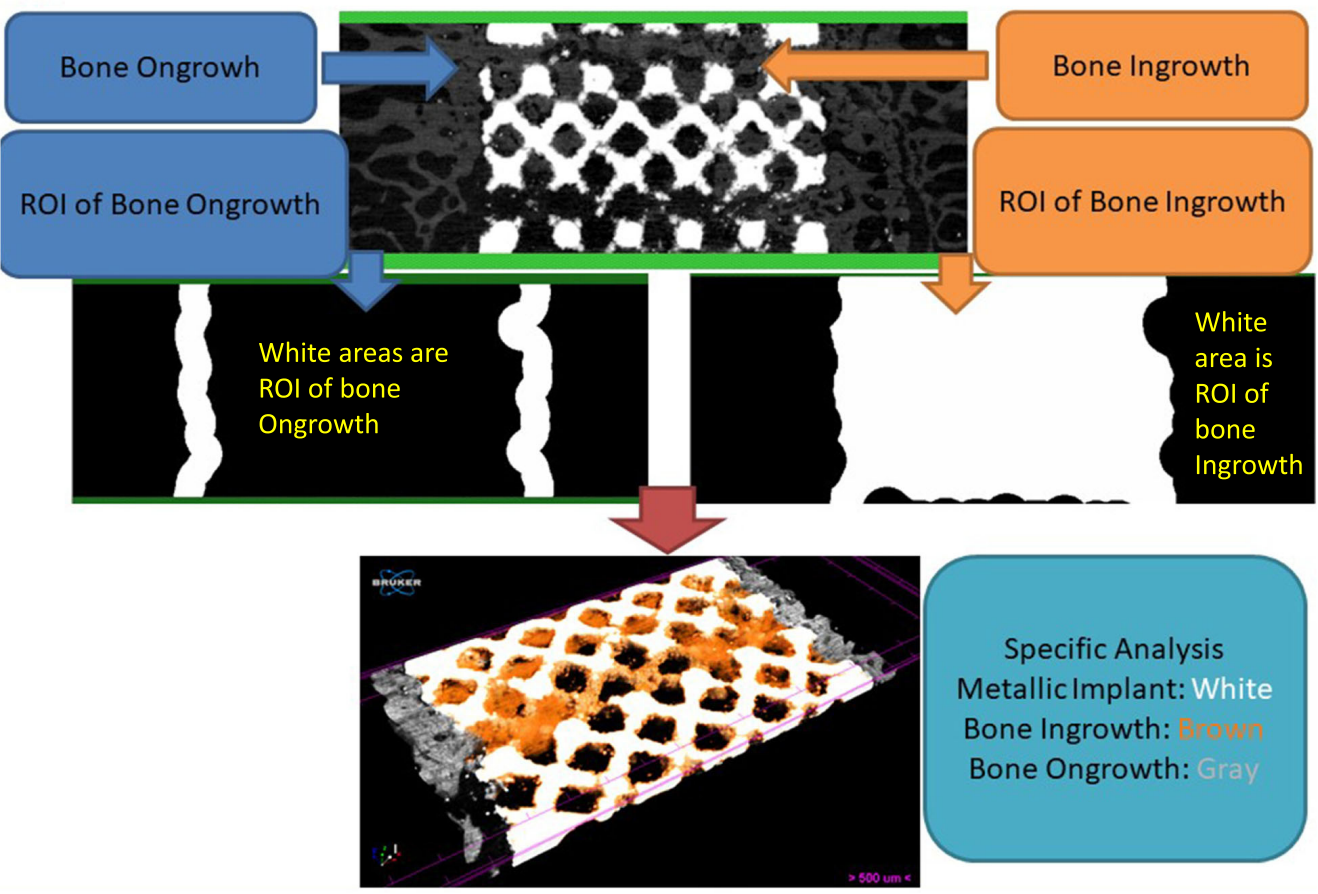
The ROIs (region of interest) of bone ongrowth analysis and bone ingrowth analysis were shown. ROI of bone ongrowth was defined as 0–500 μm around metallic implant border. ROI of bone ingrowth was defined as the area inside metallic implant border. The border was calculated by computer using shrink-warp algorithm

After euthanasia, five specimens were retrieved from each group and scanned using micro-CT (Skyscan 1272® at 8-μm/pixel, Bruker Micro-CT, Kontich, Belgium). A 360° scan with a high voltage of 90 kVp, current of 111 μA, and output of 10 W was conducted. Image reconstruction was performed using a graphics processing unit-based reconstruction software, GPU-NRecon. Ring-artifact and beam-hardening correction were also performed using GPU-NRecon. The reconstructed cross sections were reoriented, and regions of interest (ROIs) were further selected. Automatic thresholding and 3D/2D structure and pore analyses were performed using CTAn software. We performed the analysis with 1.4-mm (113 slices) images. Metallic structure and bone were separately isolated by the difference of X-ray absorption (Hounsfield Units, HU). The border of metallic structure was calculated by CTAn software using shrink-warp algorithm. ROIs of bone ongrowth was defined as 0–500 μm around metallic implant border. ROIs of bone ingrowth was defined as the area inside metallic implant border. Tissue volume (TV, mm^3^), bone volume (BV, mm^3^), percent bone volume (BV/TV, %), bone surface (BS, mm^2^) area, and bone surface area per total volume (BS/TV, 1/mm) were measured 0–500 μm above the metallic implant bone. With the nonporous implant as a template, the outer bone was defined by those exterior to the nonporous implant surface (including the bone outside the doughnut and bone of the doughnut hole), as illustrated in Fig. 2. The inner bone was defined by the bone formed interior to the surface of the implant (the doughnut body). 3D visualization was performed using Avizo software (Version 9.4, Thermo Fisher Scientific, MA, USA).

### Backscattered-Electron SEM

Three specimens were retrieved from each group and scanned using backscattered-electron SEM (BSE-SEM) at 6 months postoperatively. The specimens were decalcified before the procedure, embedded using Technovit 9100 (Kulzer, Wehrheim, Germany), and then cut into thin 1-mm slices. The slices were carefully polished and coated with carbon for BSE-SEM (DSM940; Carl-Zeiss AG, Oberkochen, Germany) analysis. Multiple images were merged using Photoshop CC (Adobe, San Jose, CA, USA). We then converted images into grayscale and analyzed them using the 2D analysis function of CTAn software (Bruker Skyscan, Konitch, Belgium). We defined the implant area as a region of interest (bone ingrowth area). Next, we expanded the bone ingrowth area by approximately 500 μm and then excluded the bone ingrowth area. The surrounding area was defined as the bone ongrowth area. Morphometric indices of ingrowth area, ongrowth area, and total area (ingrowth + ongrowth) were analyzed.

### Histological analysis

Four specimens were retrieved from each group for histological analysis at 6 months postoperatively. All these harvested samples were fixed in 10% formalin for 14 days and sequentially dehydrated with increasing concentrations of ethanol (70, 95, and 100%) for at least 1 day and infiltrated for 5 days with polymethylmethacrylate. After embedding, the samples were cut horizontally, perpendicular to the axis of bony endplates, at the level of the respective bone–implant interfaces. The sections were cut to approximately 150 μm in thickness by using an IsoMet™ Low Speed saw (Buehler, Lake Bluff, IL, USA) and ground to 60 μm with a grinding and polishing machine. The ground sections were then stained with Sanderson’s Rapid Bone Stain (Dorn & Hart Microedge Inc., Loxley, AL, USA) and then counterstained with acid fuchsin. All bone–implant interfaces were examined carefully under a light microscope. In addition, the sections were examined through fluorescence microscopy to identify new bone formation, which was labeled with tetracycline.

### Statistical analysis

All experimental data are presented as the mean ± standard deviation, with values from more than three experiments. The Wilcoxon rank sum test and Fisher’s exact test were used for nonparametric analysis. Data with more than two groups were compared through one-way analysis of variance and Tukey’s post hoc test for repeated measures. The correlation was examined as Pearson correlation and Spearman correlation; *p* < 0.05 was considered statistically significant. The power value was set to 0.8. Sample size calculation showed that the animal study required 11 in each group based on data by Zou et al. [21]. Statistical analysis was performed using PASW software (version 18.0; SPSS, Chicago, IL, USA).

## Results

### In vitro mechanical analysis of the composite cage

The Ti alloy/PEEK composite cage for interbody fusion is composed of a new hybrid material with a multilayer structure on both bony contact surfaces and a PEEK core substrate (Fig. 1a and b).

The shear strength of the interface layer of the hybrid implant was measured using an ASTM D1002 tensile test piece developed using the SLM process. As shown in Fig. 1c, when the thickness of the Ti alloy interfacial layer was 301.9 μm, the bonding strength of the laser-grooved interface layer reached 33.4 MPa. Thus, the metal–PEEK interfacial layer formed a favorable bonding structure. In addition to the shear strength test, compression and torsion mechanical tests results according to ASTM2077 were shown in Fig. 1f and g and Tables 1 and 2.

**Fig. 3:**
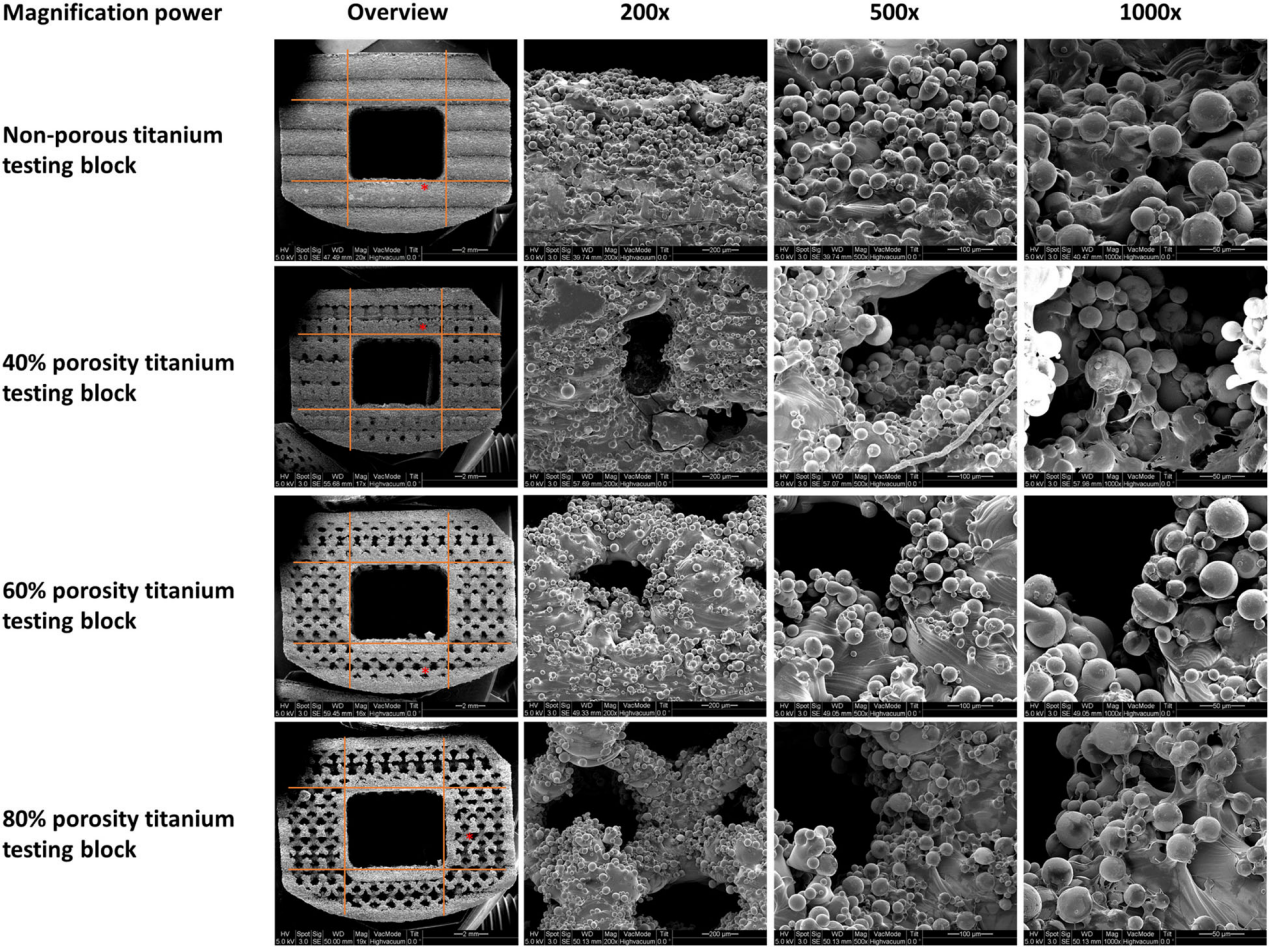
Scanning electron microscopy images of Ti-tested blocks with different porosities

The SEM analysis demonstrated superior cell growth in the higher porosity group (Fig. 3). These findings suggest that the increase in porosity from 40 to 60% increased the total surface area, and the larger surface area facilitated cell growth, differentiation, and attachment. Compared with the nonporous testing block, high-porosity cages have higher surface areas (from 40 to 60%); therefore, cells may require more time to occupy these surfaces and demonstrate growth arrest through contact inhibition.

### Micro-CT analysis

**Fig. 4:**
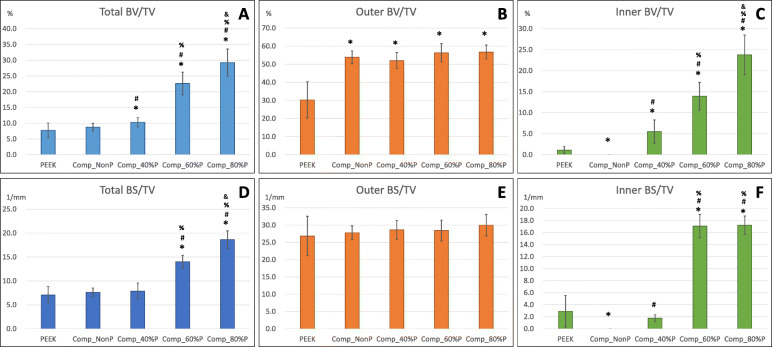
Quantitative analyses using micro-CT. **a** Total percent bone volume to total volume (total BV/TV) (%). **b** Outer percent bone volume to total volume (outer BV/TV) (%). **c** Inner percent bone volume to total volume (inner BV/TV) (%). **d** Total percent bone surface to total volume (total BS/TV) (1/mm). **e** Outer percent bone surface to total volume (outer BS/TV) (1/mm). **f** Inner percent bone surface to total volume (inner BS/TV) (1/mm). The groups were as follows: commercialized PEEK cage group (Anterior Cervical Interbody Fusion Cage®, BAUI biotech, New Taipei City, Taiwan) and composite Ti alloy/PEEK cage groups including nonporous Comp_NonP composites with 40%-, 60%-, and 80%-porosity endplates (Comp_40%P, Comp_60%P, and Comp_80%P, respectively) [* *p* < 0.05 between PEEK and other groups; # *p* < 0.05 between Comp_NonP group and other porous groups; % *p* < 0.05 between Comp_40%P and porous groups (Comp_60%P and Comp_80%P); and *p* < 0.05 between Comp_60%P and Comp_80%P groups]

**Fig. 5:**
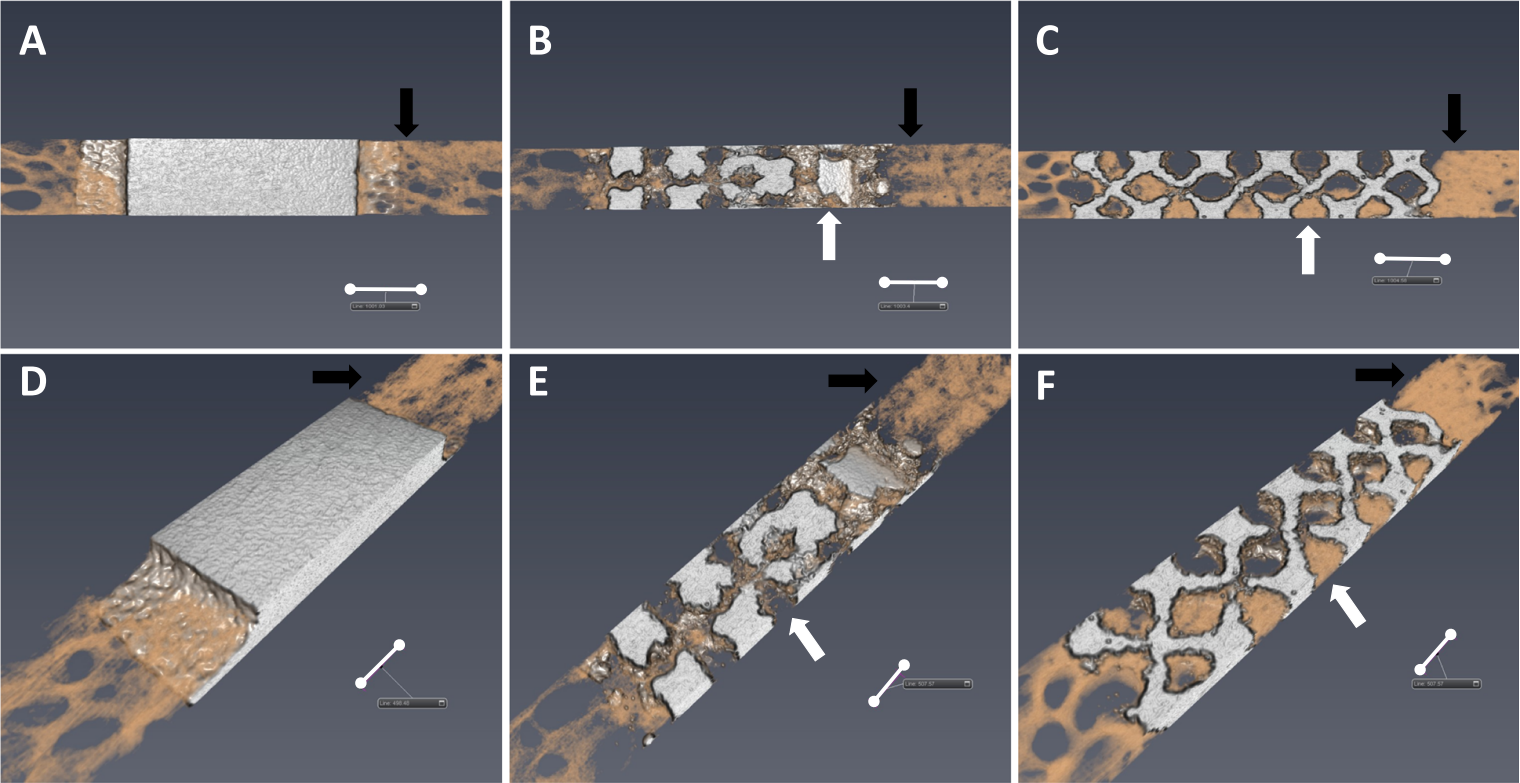
Representative images of micro-CT. Comp_NonP (**a** and **d**), Comp_60%P (**b** and **e**), and Comp_80%P (**c** and **f**). The black arrows indicate bone ongrowth and white arrows indicate bone ingrowth. Scale bar = 1 mm

Micro-CT was used to evaluate bone formation between the implant and bone tissue. Compared with PEEK and nonporous cages, the porous composite cage (irrespective of the porosity rate) demonstrated significantly higher total and outer BV/TV at 6 months postoperatively at the bone–implant interface (Fig. 4a and b). This finding suggests that the Ti implant recruited more bone than did PEEK. However, the inner BV/TV significantly increased with the porosity rate (Fig. 4c). These findings suggest that the increased porosity rate resulted in increased bone formation within the implant but not outside the implant (Fig. 5b, c, e, and f).

The bone surface density (BS/TV) represents the bone at the surface of the implant. Higher BS/TV suggests more bone growth close to the defined implant surface area. As shown in Fig. 4d–f, composite cages with 60 and 80% porosity exhibited significantly higher total and inner BS/TV than did the other groups, suggesting that most of the formed bone was close to the porous structure (inside the implant) rather than outside the implant.

**Fig. 6:**
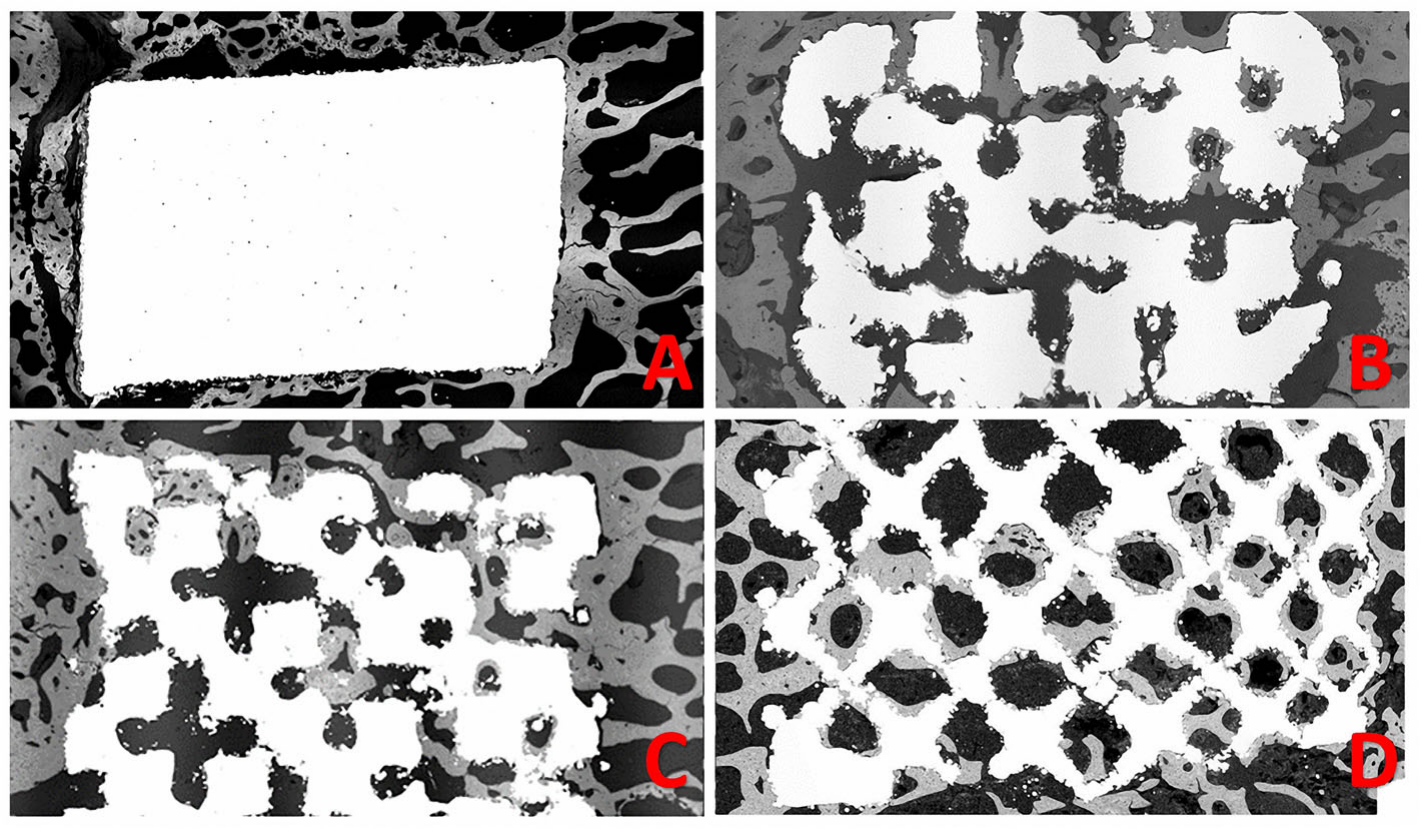
Representative backscattered-electron scanning electron microscopy images of Comp_NonP (**a**), Comp_40%P (**b**), Comp_60%P (**c**), and Comp_80%P (**d**)

BSE-SEM analysis revealed results similar to those obtained from 3D micro-CT analysis (Fig. 6).

In summary, our micro-CT and BSE-SEM results demonstrated that structures with higher porosity, especially those with 60 and 80% porosity, facilitated more bone formation inside the implant; however, differences in bone formation outside the implants were nonsignificant between the groups.

### Histological analysis

**Fig. 7:**
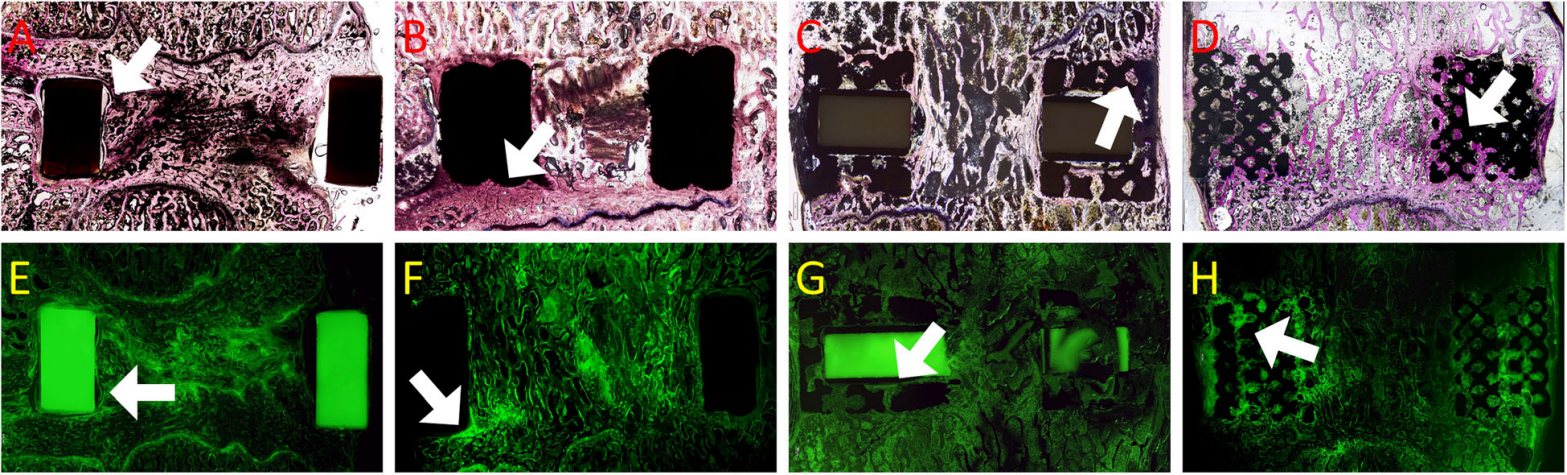
Histological analysis and fluorescence microscopy. Sections **a**–**d** were stained with Sanderson’s Rapid Bone Stain and counterstained with acid fuchsin (RBS). Sections E–H were examined with fluorescence microscopy to identify new bone formation labeled with tetracycline. A and E: PEEK cage; B and F: Ti alloy nonporous cage; C and G: Ti alloy/PEEK composite cage with 60% porosity; and D and H: Ti alloy/PEEK composite cage with 80% porosity. A and E: white arrows indicate a gap between the bone and implant. B and F: white arrows denote close contact between the bone and implant, with new bone formation on the interface. C, D, G, and H: white arrows represent bone growth into the porous structure of the implant

Compared with Ti alloy groups, bone formation at the bone–implant interface was lower in the PEEK group (Fig. 7a and b). Similar results were obtained from tetracycline-labeled bone fluorescence microscopy (Fig. 7e and f). The gap between the two surfaces was larger in the PEEK group than in the Ti alloy group. As shown in Fig. 7a and e, bone formation appeared to encapsulate the implant rather than grow inside the implant. By contrast, the Ti alloy group had superior bone and implant contact; the bone and implant gap was much smaller and sometimes even difficult to identify (Fig. 7b and f).

Histological analysis (Fig. 7c and d) as well as tetracycline-labeled bone fluorescence microscopy (Fig. 7g and h) revealed bone growth on the porous structure.

**Fig. 8:**
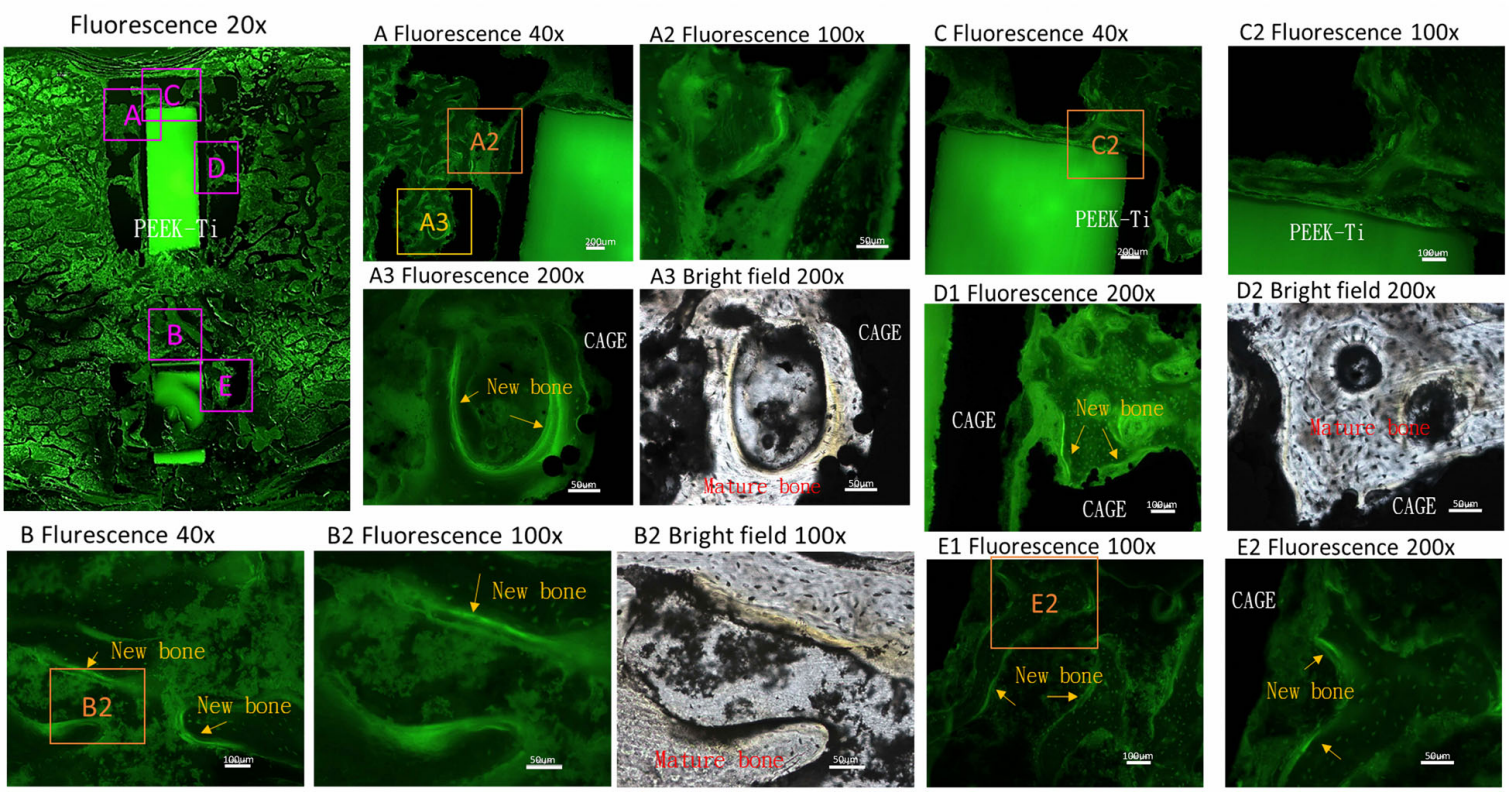
Histological analysis of bone sections by using tetracycline through fluorescence and bright-field microscopy. New bone formation was easily identified inside the porous structure of the implant (Pig No. 14, L1/2, Ti alloy/PEEK composite cage with 60% porosity). Areas are picked-up by observed obvious bone ingrowth

Figures 7 and 8 show tetracycline-labeled new bone growth into the space of the porous Ti structure, indicating peri-implant osteogenesis. However, because of the qualitative nature of the histological analysis, we were unable to compare the amount of bone formation between different porosity rates.

In summary, bone formation in the Ti alloy groups was vastly superior to that in the PEEK group, with 60 and 80% porosities being most beneficial for bone formation.

## Discussion

We developed a new porous Ti-6Al-4 V/PEEK composite interbody cage. The innovative interfacial construction resulted in a high shear strength of more than 30 MPa of the bonding interface between the Ti alloy and PEEK substrate. This shear strength is much higher than those previously reported [7, 9, 13, 19]. In addition, different Ti alloy porous structures can be easily manipulated above the solid interface layer in the additive manufacturing process to meet individual requirements.

PEEK cages have been widely used for spinal fusion operations [9, 16, 18, 22]. Their advantages include radiolucency and low elastic modulus [9, 14, 16, 18]. Our experimental results are highly compatible with those of previous studies examining PEEK cages packed with the iliac bone graft [18, 23, 24]. However, PEEK cages have exhibited inferior implant–host bone growth because of their chemically inert character, resulting in pseudarthrosis, nonfusion, implant migration, and subsidence [7, 18, 25]. These shortcomings can be overcome by coating bioactive substances at the bone-contact surface layers of PEEK cages while maintaining their advantages [7, 12, 13]. Ti alloy is one of the most common metals used with PEEK cages [26], and porous Ti implants create an osteoconductive environment by providing not only immediate stability resulting from interfacial friction but also long-term bony ongrowth and ingrowth [7, 25].

In the present study, in vivo experiments demonstrated favorable bone growth on Ti implants. Higher porosity rates led to larger total surface area and facilitated larger amount of bone formation within the porous structure, new bone formation can also be clearly identified in the porous structure. In addition, SEM analysis demonstrated superior cell growth in the higher porosity group.

In the porcine model, significantly superior bone ingrowth into porous-structure composite cages was observed. Compared with the PEEK and nonporous Ti alloy groups, the porous composite cage groups demonstrated superior bone growth in micro-CT and histological analyses. According to micro-CT analysis, the BV/TV was significantly higher in the high-porosity (60 and 80%) groups than in the PEEK and nonporous groups, indicating greater bone volume accumulation (bone growth) in the high-porosity composite cage group. More specifically, the main difference was derived from the bone growth into the implant; this is evidenced by the significantly higher inner BV/TV in the 60%- and 80%-porosity groups. By contrast, differences in outer BV/TV between the groups were nonsignificant. In addition, we compared the BS/TV between the groups. Compared with BV/TV, BS/TV more directly indicates bone accumulation closer to the implant surface. The results clearly demonstrated significantly higher total and inner BS/TV in the high-porosity groups (60 and 80%) than in the PEEK and nonporous groups, but the outer BS/TV in each group was not significantly different. All these findings suggest that the majority of bone growth in the porous groups was into the implant instead of at the outermost surface of the implant. Moreover, the bone growth close to the implant was superior on the Ti alloy surface to that on the PEEK surface.

Histological analysis results revealed new bone formation with calcium deposition within the porous structure of the composite cage. For the PEEK implant, the gap between the implant and bone was larger than that for Ti alloy implants. This result is compatible with a previous finding that the hydrophobic surface property of PEEK makes protein absorption difficult and results in poor cell adhesion and bone growth [17]. By contrast, the Ti alloy cage demonstrated close contact between the implant and bone. Even in the absence of a porous structure, clearly identifying the gap between the bone and implant interface was difficult. For Ti alloy/PEEK composite cages with a porous structure, bone growth into the porous structure was clearly identified. As shown in Fig. 8, tetracycline-labeled new bone growth into the space of the porous Ti structure clearly indicated peri-implant osteogenesis. Peri-implant osteogenesis is a multistep process that includes osteoblast adhesion, proliferation, and differentiation and involves the production of specific proteins and deposition of calcium phosphate in the extracellular matrix [7, 27]. Our histological analysis demonstrated osteogenic incorporation into the porous structure of the porous composite cage. This result explains a previous micro-CT analysis finding of a much larger inner BV/TV and BS/TV for porous cages. In summary, bone ingrowth into the porous composite cage enhanced host-bone implant incorporation.

Although our study provided promising results, it has some limitations. First, the study was not performed in an upright vertebral system. An in vitro mechanical test in our previous work showed sufficient mechanical strength of the composite implant [20], and no mechanical failure was noted in the present porcine study. However, future clinical studies should validate the mechanical performance of the composite implant in the upright vertebral system. Second, our results revealed the optimal compromise between mechanical strength and bone growth when the Ti alloy/PEEK composite cage with 60% porosity was used. However, whether 60% porosity is the optimal condition is unclear, and more detailed analyses are required to identify the optimal porosity for bone growth and mechanical strength. Third, because the porous structure was designed symmetrically through mathematical calculations, it was unclear whether different porous structures affected bone growth.

## Conclusion

The present study clearly demonstrated that the porous Ti alloy endplate of the composite cage facilitated bone ongrowth and ingrowth and that the central PEEK portion reduced the elastic modulus and presented the clinical advantage of radiolucency. In addition, the innovative interfacial bonding layer exhibited sufficient mechanical strength for clinical application. The composite cage implant combined the advantages of the biological properties of porous Ti alloy endplates and biomechanical and radiographic properties of central PEEK, which makes it a suitable solution for intervertebral fusion surgery and can be further developed for clinical use.

## Supplementary Information

**Additional file 1.**

**Additional file 2.**

**Additional file 3.**

**Additional file 4.**

**Additional file 5.**

**Additional file 6.**

**Additional file 7.**

**Additional file 8.**

**Additional file 9.**

## Data Availability

The datasets generated and/or analyzed during the current study are not publicly available because they contain trade secrets but can be made available from the corresponding author on reasonable request.

## Abbreviations

PEEK: Polyetheretherketone
Ti: Titanium
Ti-6Al-4 V: Titanium alloy
CT: Computed tomography
BSE-SEM: Backscattered-electrons scanning electron microscope
TV: Tissue volume
BV/TV: Bone volume, percent bone volume
BS: Bone surface
BS/TV: Bone surface area per total volume
