# Supplementary figures and images for: Additive-manufactured Ti-6Al-4 V/Polyetheretherketone composite porous cage for Interbody fusion: bone growth and biocompatibility evaluation in a porcine model
Source: BMC Musculoskelet Disord. 2021 Feb 11;22:171. doi: 10.1186/s12891-021-04022-0 (PMC7879644; doi:10.1186/s12891-021-04022-0)

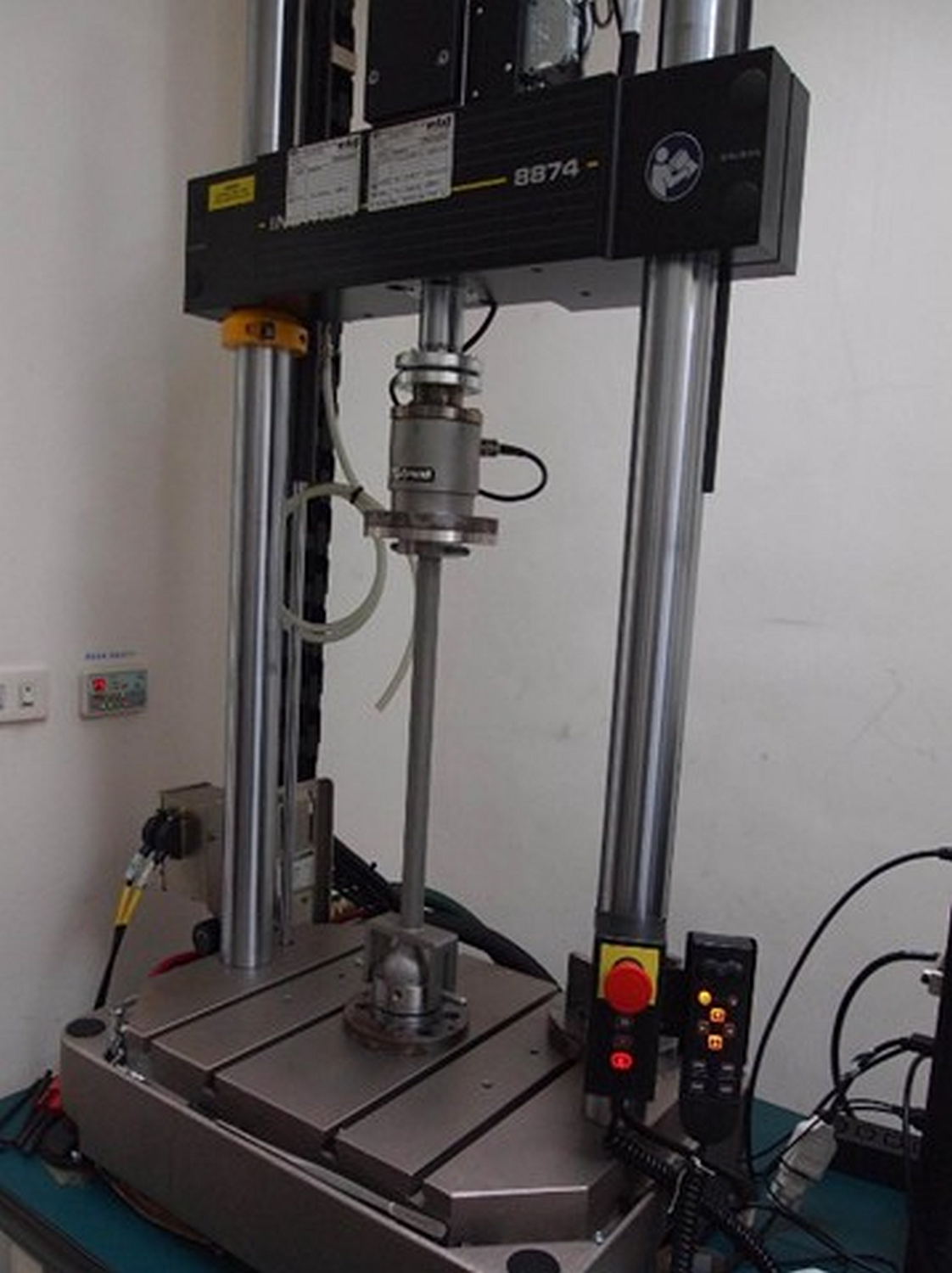

Supplement: Supplementary file 1 — Additional file 1. [file 12891_2021_4022_MOESM1_ESM.tif]

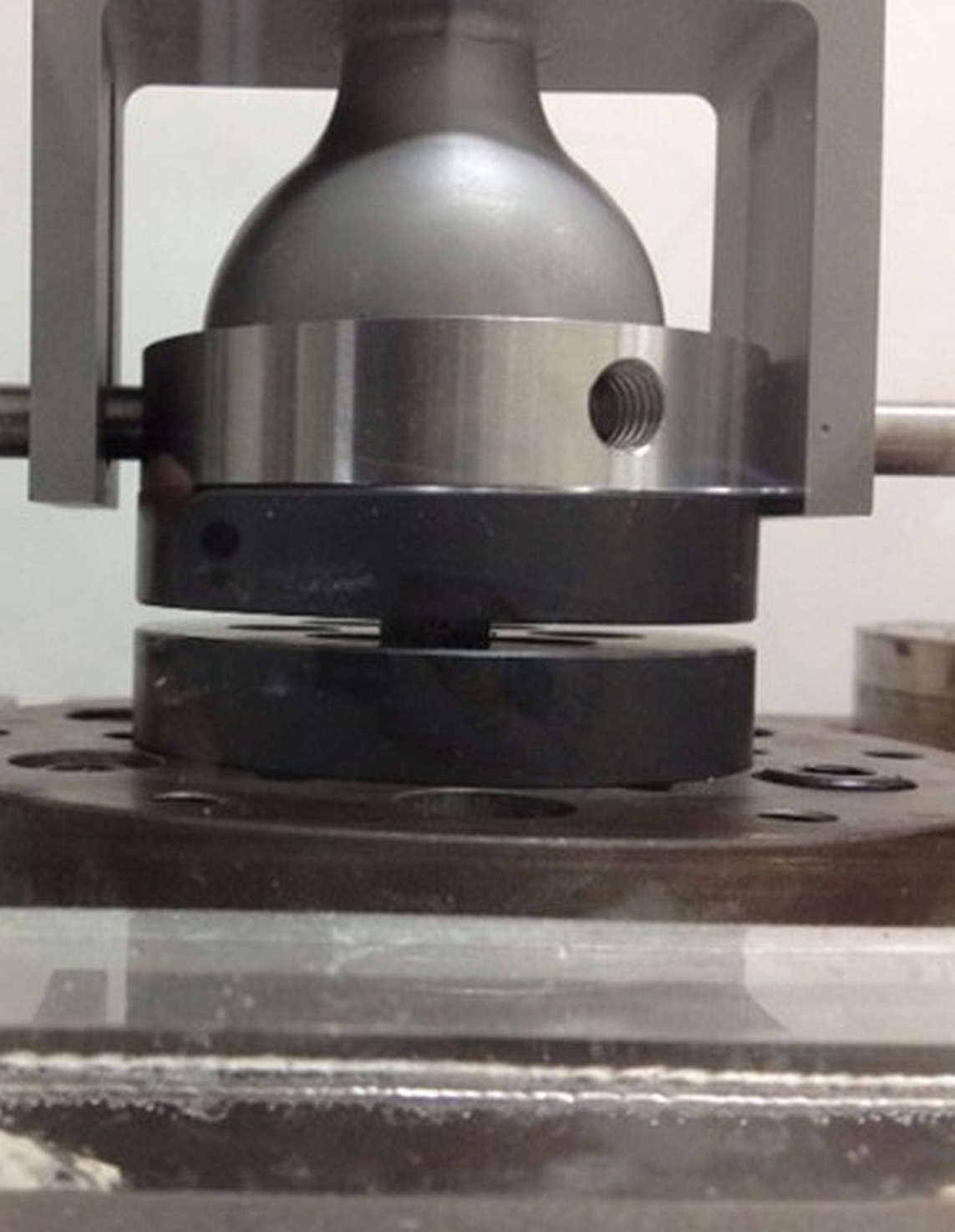

Supplement: Supplementary file 2 — Additional file 2. [file 12891_2021_4022_MOESM2_ESM.tif]

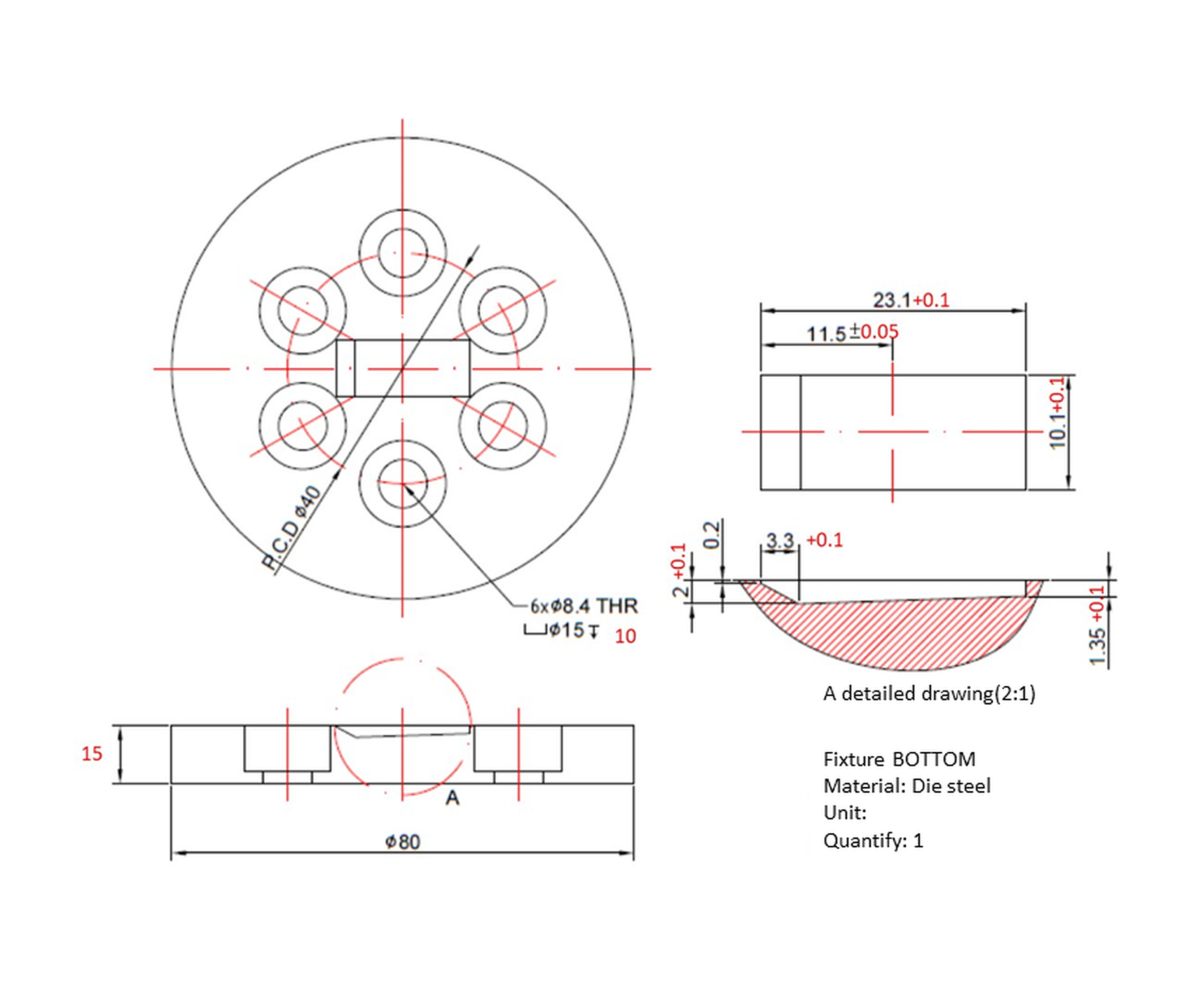

Supplement: Supplementary file 3 — Additional file 3. [file 12891_2021_4022_MOESM3_ESM.tif]

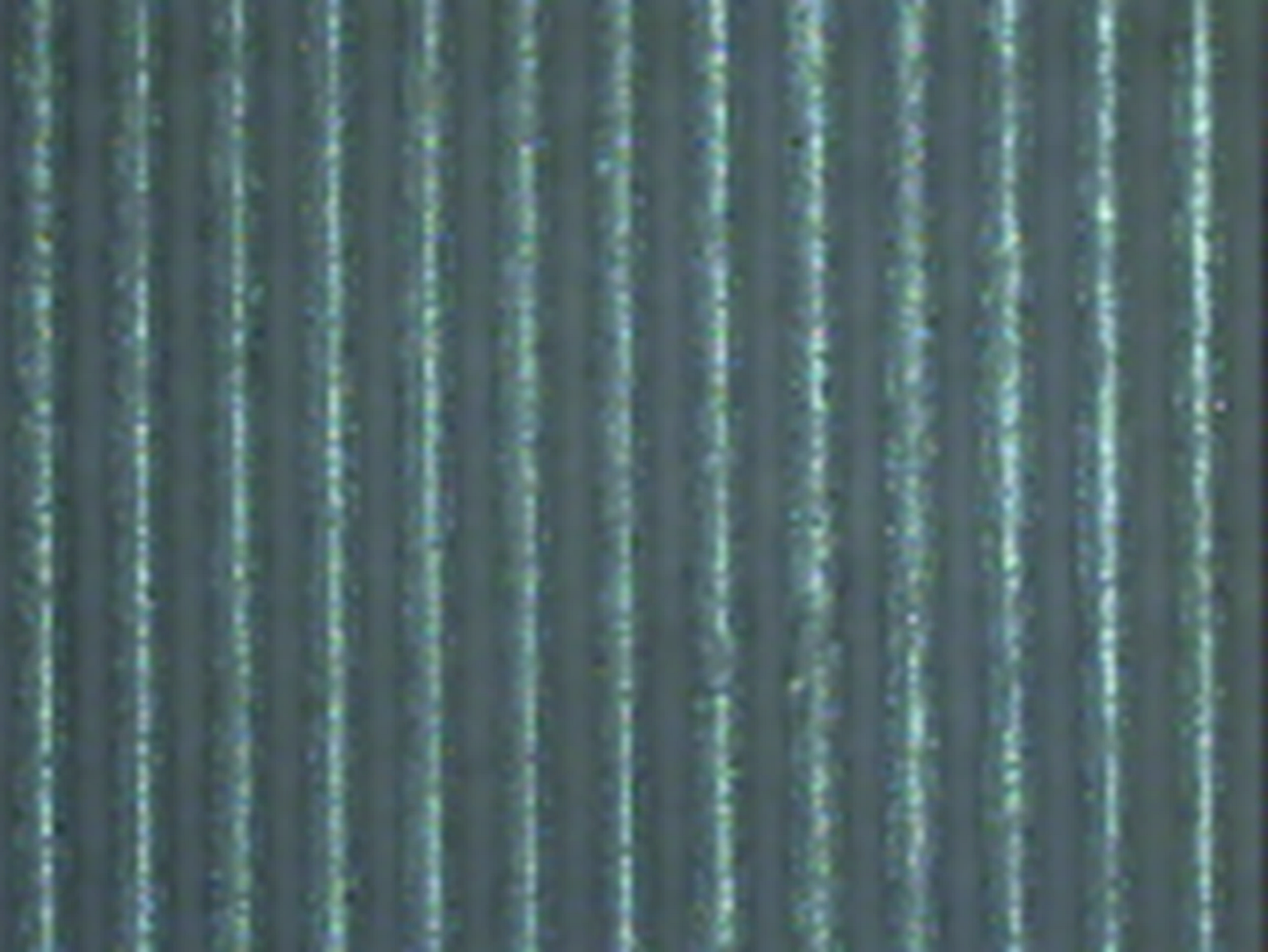

Supplement: Supplementary file 4 — Additional file 4. [file 12891_2021_4022_MOESM4_ESM.tif]

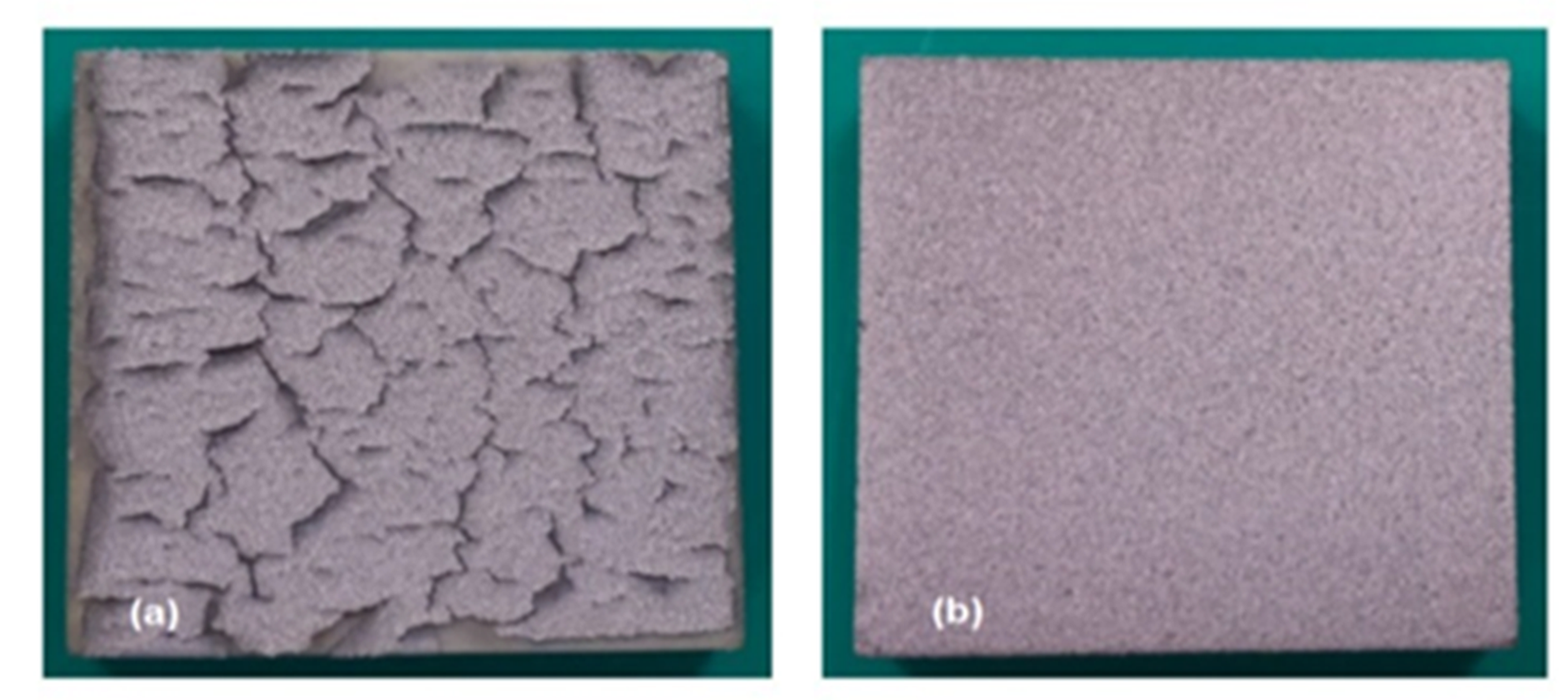

Supplement: Supplementary file 5 — Additional file 5. [file 12891_2021_4022_MOESM5_ESM.tif]

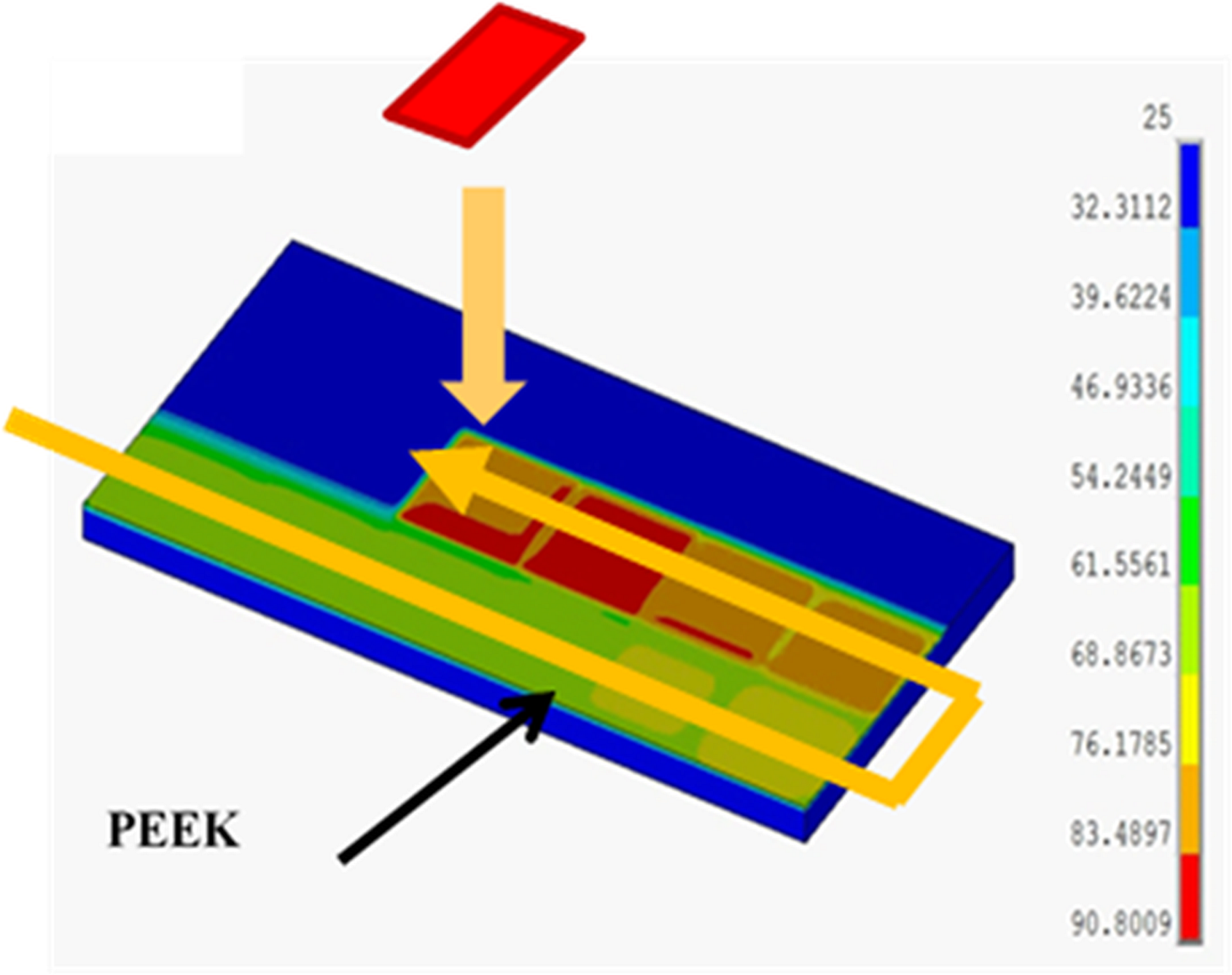

Supplement: Supplementary file 6 — Additional file 6. [file 12891_2021_4022_MOESM6_ESM.tif]

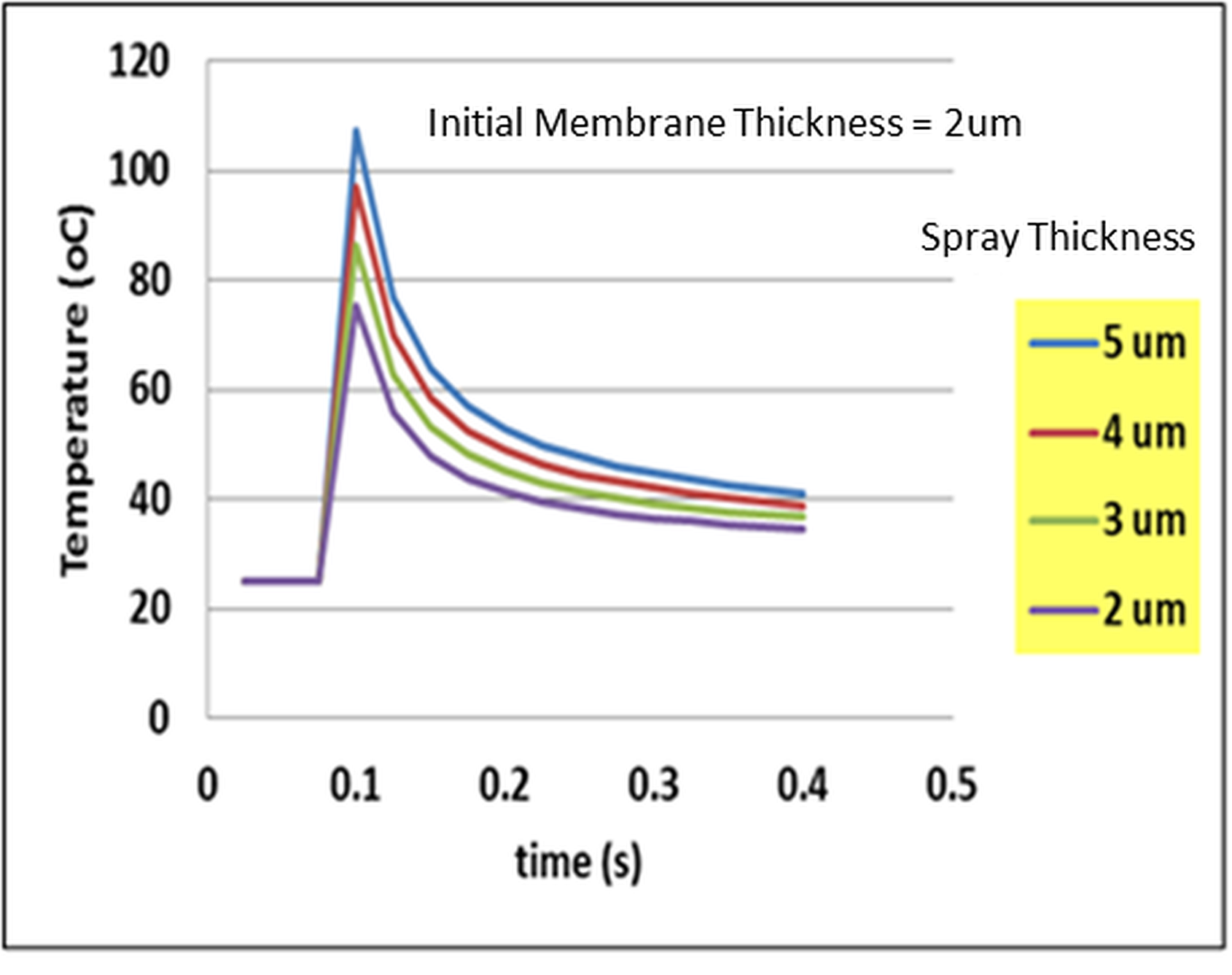

Supplement: Supplementary file 7 — Additional file 7. [file 12891_2021_4022_MOESM7_ESM.tif]

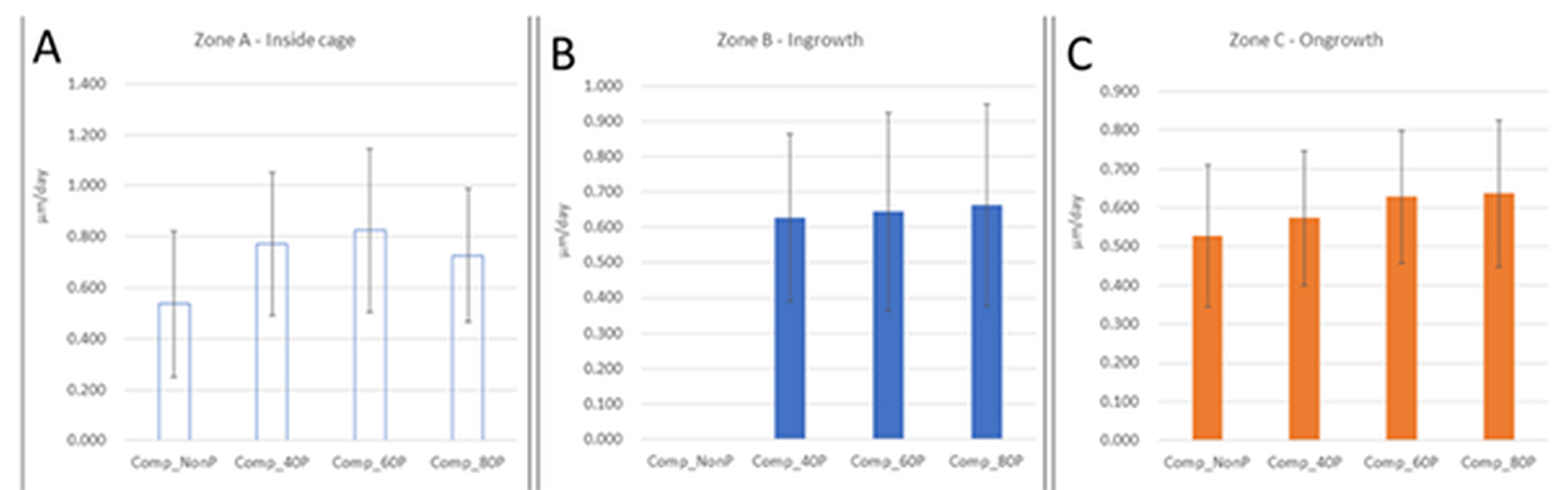

Supplement: Supplementary file 8 — Additional file 8. [file 12891_2021_4022_MOESM8_ESM.tif]

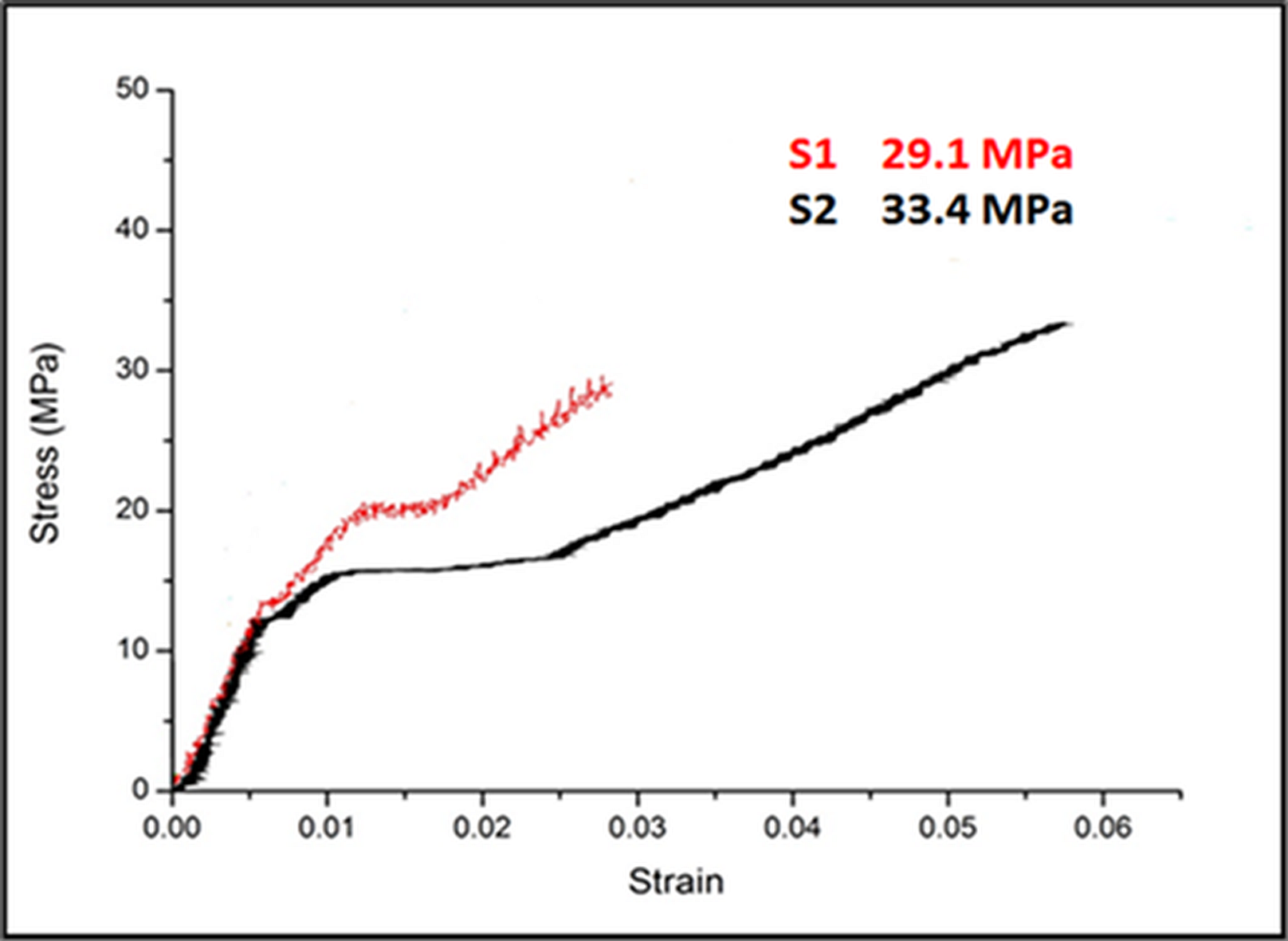

Supplement: Supplementary file 9 — Additional file 9. [file 12891_2021_4022_MOESM9_ESM.tif]
